# Reliability and validity of two hand dynamometers when used by community-dwelling adults aged over 50 years

**DOI:** 10.1186/s12877-022-03270-6

**Published:** 2022-07-15

**Authors:** Li Huang, Yadong Liu, Taiping Lin, Lisha Hou, Quhong Song, Ning Ge, Jirong Yue

**Affiliations:** Department of Geriatrics and National Clinical Research Center for Geriatrics, West China Hospital, Sichuan University, Sichuan Province, Chengdu, 610041 China

**Keywords:** Hand dynamometer, Muscle strength, Sarcopenia, Reliability and validity

## Abstract

**Background:**

The Jamar hydraulic dynamometer is a widely recognized tool for measuring grip strength. Nevertheless, the devices used most often in Asian countries are spring-type dynamometers, represented by the CAMRY dynamometer or Smedley dynamometer. We aimed to evaluate the reliability and validity of the CAMRY dynamometer compared with the Jamar dynamometer.

**Methods:**

This was a cross-sectional study using a random crossover design in the grip strength test with two dynamometers. A total of 1064 healthy community-dwelling older adults aged 50–90 years old, which included 686 minorities and 378 Han Chinese, were recruited into the study from July to September 2021. We assessed the reliability and validity of the CAMRY EH101 dynamometer, and the Jamar dynamometer was regarded as the reference device. The order of testing with two dynamometers was randomized in a 1:1 ratio, with a 10-min gap between the two devices. Intraclass correlation coefficients (ICCs) and Bland–Altman analysis were calculated to assess reliability and validity between the two devices.

**Results:**

The average handgrip strength (HGS) values at six times by the Jamar and CAMRY devices were 25.0 ± 7.9 kg and 24.6 ± 7.5 kg, respectively. The ICC values between the two devices were 0.815–0.854, and the systematic bias underestimated by the CAMRY dynamometer was 0.5 kg in men and 0.6 kg in women. We carried out a linear regression equation by sex, and their relationship was found as follows: male HGS (kg)_Jamar_ = 8.001 + 0.765 × HGS (kg)_CAMRY_; female HGS (kg)_Jamar_ = 3.681 + 0.840 × HGS (kg)_CAMRY_.

**Conclusions:**

The CAMRY EH101 dynamometer provides excellent reliability and validity. This device can serve as a reliable, inexpensive, and practical device to assess grip strength in geriatric clinical practice.

**Clinical trial registration:**

Chinese Clinical Trial Registry: ChiCTR2100046367; Date of clinical trial reistration: 15/05/2021.

## Introduction

Muscle weakness is a marker of various poor health outcomes, such as bone health, cardiometabolic disease risk, physical dysfunction, and all-cause mortality [1]. Muscle strength is a crucial component to diagnose sarcopenia and frailty [2]. Previously published studies have reported several muscle strength instruments and procedures, including handgrip dynamometers, isokinetic dynamometers, elastic bands, leg press, pull down and vigorimeter [3]. In fact, the isokinetic dynamometer is difficult to move and operate, and elbow flexion and knee extension are limited by requiring special equipment and training. The handgrip dynamometer is the widely recommended device to measure muscle strength in the Asian Working Group for Sarcopenia (AWGS) 2019 [4].

**Fig. 1:**
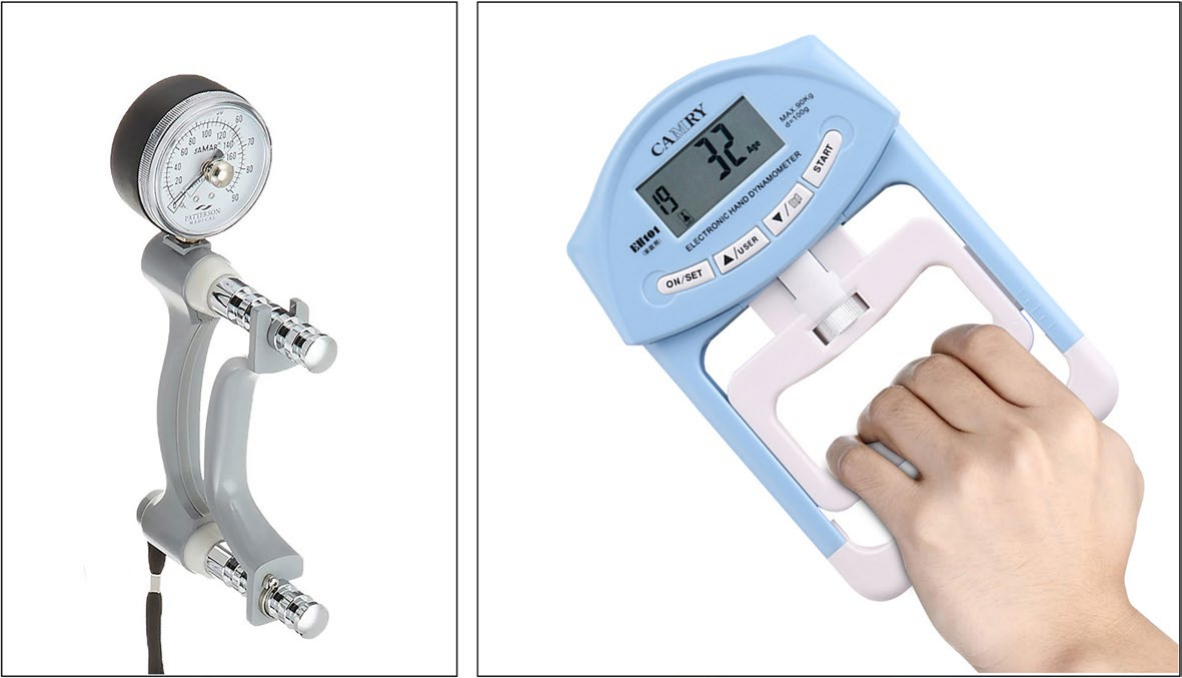
Jamar dynamometer (left) and CAMRY EH101 dynamometer (right)

Instruments for measuring HGS can be divided into four classes [5] according to physical principles: (a) Hydraulic dynamometer: a Jamar hydraulic dynamometer is a typical representative; grip strength is determined by hydraulic conduction. (b) Pneumostatic dynamometer: grip strength is assessed according to the grip pressure change by squeezing the rubber bulb, such as a Martin vigorimeter. (c) Spring-type dynamometer: The spring-type dynamometer is the widely recommended device to measure handgrip strength in Asian countries [4] and is simple, inexpensive and accurate. The CAMRY dynamometer (CAMRY EH101, Sensun Weighing Apparatus Group Ltd, Guangdong, China) is widely used in China, and the Smedley dynamometer (Smedley YD-100, Tokyo, Japan) is widely used in Japan and other countries. (d) Strain hand dynamometer: grip strength measurement is based on the variation in electrical resistance of a length of wire due to strain applied to it. The isometric strength testing unit is a representative device to measure whole-body static strength in various positions [6]. The Jamar dynamometer was invented in 1954 and can record grip strength values with five different handle positions [7]. This device is considered the gold standard by which other dynamometers are compared because it has the highest retest reliability and precision. Comprehensive application of the Jamar dynamometer (the left of Fig. 1) is limited in China due to the high price, rare purchase channels, and constant maintenance. The CAMRY dynamometer (the right of Fig. 1) is widely used in the majority of health care settings and physical training for middle school students.

The Asian expert consensus on sarcopenia recommended using spring-type dynamometers or hydraulic-type dynamometers to diagnose sarcopenia. Different dynamometers may have different absolute values even for the same person. Kim et al. [8] compared Jamar and Smedley dynamometers in 467 community-dwelling older adults aged 69–89 years and found a statistically significant difference between them despite the high correlation. Interestingly, the CAMRY dynamometer and Smedley dynamometer share the same physical principle and operation procedure. To date, no studies have explored whether JAMAR hydraulic dynamometers and CAMRY spring dynamometers are consistent, let alone calculated the difference bias between them. The present study aimed to explore the relationship between the Jamar hydraulic-type and CAMRY spring-type dynamometers in a large community-dwelling older Chinese population. The primary purpose was to assess the reliability and measurement bias of grip strength obtained with CAMRY EH101 dynamometers. The second objective was to calculate the simplified equation between the two dynamometers.

## Methods

### Participants

Our participants came from an existing longitudinal population-based community cohort study, the West China Health and Aging Trends Study (WCHAT), and the criteria for inclusion in the cohorts were people aged over 50 years. The initial intent of the WCHAT cohort was to establish a sample biobank in relation to the risk and prognosis of frailty, sarcopenia, and other geriatric syndromes. Our study aimed to evaluate the reliability and validity of the CAMRY dynamometer, and all participants performed handgrip strength measurements by two dynamometers simultaneously. Participants underwent sarcopenia and frailty screening in this research. Since the purpose of this study did not include screening for sarcopenia and frailty, we did not report it specifically.

To further explore the relationship between the two dynamometers used by different populations, we recruited both ethnic minorities who lived in the Tibetan Qiang Autonomous Prefecture in Sichuan Province and Han Chinese who lived in Chengdu in Sichuan Province. Tibetan Qiang Autonomous Prefecture in Sichuan Province is a plateau region at an altitude of 2000 m above sea level. People living there are Qiang, Tibetan and Yi ethnic minorities. The inclusion criteria of this study were as follows: (a) regular medical check-ups and good activity capacity; (b) compliance, defined as being serious about completing the study. Participants with fractures, trauma, deformities, acute exacerbation of joint diseases, rheumatoid disease, and gout, neuromuscular disease or other acute conditions affecting the grip strength test were excluded from this study. Individuals with unstable chronic disease or other conditions that may affect the hand functional examination were also excluded.

Finally, 1064 older adults aged 50–90 years, including 686 minorities and 378 Han Chinese, were enrolled from July to September 2021. The study was performed in accordance with the Declaration of Helsinki and approved by the Institutional Review Boards of West China Hospital, Sichuan University; the approval number is 2021(96). Written informed consent was obtained from all participants (the participants who were unable to write by providing a thumb print and the authorization letter were also signed by the legal guardians).

### Grip strength measurements and procedures

First, two professional researchers explained how to use the two dynamometers and inquired about hand dominance. Dominance is defined as carrying out well-learned skills such as writing, holding chopsticks and throwing a ball. Generally, dominance is right-handed for the majority of people. Three grip strength tests were performed with two dynamometers for each hand, with a 10-min gap between the two devices. The test order of the two dynamometers was randomized in a 1:1 ratio, and randomization was carried out according to an Excel random number table. The second handle position has been assumed to be the most reliable position because it can help to maximize grip strength [5]. Both the Jamar dynamometer and CAMRY handgrip dynamometer were set in the second-handle position. Standard testing procedures of the Jamar hydraulic dynamometer are listed as follows: the subjects were seated in a comfortable chair without arm support, with the elbow in 90° flexion; the upper arm and lateral thorax were separated to ensure accuracy. This position is also recommended as a standardized grip strength testing guideline by the American Society of Hand Therapists (ASHT). Measurement of the CAMRY dynamometer was performed with the elbow fully extended in the standing position. In this research, grip strength testing was performed on two devices using a standardized testing protocol. Subjects were asked to squeeze the handle with maximal effort for at least 5 s and were given verbal encouragement. A break of at least 15-s was taken between the two tests to prevent fatigue effects. Both hands were tested three times with maximal effort, and average HGS values were mainly recorded and analysed. HGS values were measured in kilograms, and a kilogram was equal to 2.2046 pounds. The maximum value measured by the CAMRY dynamometer is 90 kg, and the limitation of accuracy is 0.1 kg. All participants strictly carried out HGS testing procedures under the supervision and instruction of two specialized researchers. A standard operation procedure (SOP) was developed in this study, and data were deposited into the unified database platform. Five research staff checked the data and treated missing values according to age and sex.

### Other anthropometric measurements

The elements of anthropometry include height, weight, mid-upper arm circumference (MUAC), calf circumference (CC), waist circumference, hip circumference, blood pressure and pulse. Height and weight in this study were measured by the Tsinghua Tongfang height and weight tester. Participants were asked to remove shoes and heavy clothing before the height and weight measurements. Height and weight were measured twice, and the average of two measurements was taken for analysis. The MUAC was measured at the middle point of the upper arm on the dominant side. The midpoint of the acromion and olecranon was marked when the subject was in a stand position; subsequently, the researcher wrapped the measuring tape at the marked midpoint. CC measurement was performed with the subjects in a seated position. The knee and ankle bent at 90°, and a specialized researcher used tape located at the maximum horizontal distance around the calf of the right leg. Waist circumference and hip circumference were measured with the subjects in a standing position. A trained observer measured the waist circumference of the subject at the umbilicus level after deep expiration. Hip circumference was measured with a tape measure at the largest circle level of the hip while standing. Blood pressure and pulse were measured after a 5-min rest period in a seated position using an electronic sphygmomanometer.

### Statistical analysis

Data analysis was performed with IBM SPSS (version 21.0), and the average grip strength value of six times was selected for further analysis. Study population baseline characteristics are presented as the mean ± standard deviation (SD). The reliability and measurement bias of the CAMRY dynamometer were assessed to determine accuracy and agreement with the Jamar device.

Relative reliability was assessed using the intraclass correlation coefficient (ICC). ICC was performed based on a single measurement, absolute agreement, and two-way random-effects model. Generally, ICC is considered good for 0.75–0.90 and excellent for 0.91–1.00 [9]. Absolute reliability includes the standard error of measurement (SEM) and minimal detectable change (MDC). SEM and MDC were calculated with the following formulas:

SEM = SD × $\sqrt{1-\mathrm{ICC}}$; MDC = 1.96 × $\sqrt{2}$×SEM; SEM% = (SEM/mean) × 100% [10]; MDC% = (MDC/mean) × 100% [11] *(SD: standard deviation of the difference; mean: average grip strength values of two dynamometers)*. Moreover, Spearman correlation and simple linear regression were also performed to assess correlations between the two devices. We considered Spearman correlation coefficients larger than 0.80 to be excellent.

The overall difference between the two dynamometers was determined through systematic bias and measurement error. Paired t tests and Bland–Altman analyses were performed to quantify the measurement bias of both dynamometers [12]. Systematic bias was expressed as the mean difference between two methods by Bland–Altman plots. The 95% limits of agreement (LOA) were defined as bias ± 1.96 SD, and SD was the standard deviation of the difference [13]. p < 0.05 indicated statistical significance.

## Results

### Participants and clinical characteristics

A total of 1064 healthy community-dwelling adults aged 50–90 years completed this study, which included 686 minorities living in highland areas and 378 Han Chinese individuals. The study participants consisted of 693 females and 371 males with a mean age of 66 ± 7.7 years old. A demographic description of the sample is shown in Table 1. Among the 1064 participants, 121 had sarcopenia, and 943 had nonsarcopenia. Table 2 shows the results of average grip strength and maximum grip strength in different study populations. The average HGSs of the six times by Jamar and CAMRY were 25.0 ± 7.9 kg and 24.6 ± 7.5 kg, respectively. Two dynamometers consistently showed that minorities had significantly greater HGS than Han Chinese; similarly, HGS in males was higher than in females (Table 2). Overall, HGS values estimated by the Jamar dynamometer were higher than those estimated by the CAMRY dynamometer.

**Table 1:** Baseline characteristics of sample

Characteristics	Total(*n* = 1064)	Female(*n* = 693)	Male(*n* = 371)
Ages (years)	66 ± 7.7	64 ± 7.4	68 ± 7.6
Height (cm)	155 ± 7.7	151 ± 5.7	162 ± 5.9
BodyWeight (kg)	61 ± 9.9	59 ± 9.4	65 ± 9.8
Mid-upper arm circumference (cm)	28 ± 3	28 ± 3	27 ± 3
Calf circumference (cm)	34 ± 3	34 ± 2.8	34 ± 3.3
Waist circumference (cm)	87 ± 10	87 ± 10	88 ± 97
Hip circumference (cm)	95 ± 7.2	95 ± 7	95 ± 7.5
Blood Pressure (mmHg)	135/84	135/84	136/85
Pulse (beats/min)	74	75	73
Sarcopenia (n)	121 (11.37%)	69	52
Non Sarcopenia (n)	943 (88.63%)	624	319
Chronic diseases (n)	772 (73.8%)	510 (73.5%)	262 (70.6%)
Hypertension (n)	354 (33.8%)	229 (33%)	116 (31.2%)
Coronary heart disease (n)	55 (5.2%)	32 (4.6%)	23 (6.1%)
Diabetes mellitus (n)	133 (12.7%)	93 (13.4%)	40 (10.7%)
chronic obstructive pulmonary disease (n)	72 (6.8%)	31( 4.47%)	41 (11%)
Gastrointestinal disease (n)	92 (8.7%)	52 (7.5%)	40 (10.7%)
Liver disease (n)	83 (7.9%)	54 (7.7%)	29 (7.8%)
Kidney disease (n)	52 (4.9%)	34 (4.9%)	18 (4.8%)
Cerebrovascular disease (n)	44 (4.2%)	28 (4%)	16 (4.3%)

**Table 2:** Handgrip strength of different subgroups

Handgrip strength (kg)	Male (*n* = 371)	Female (*n* = 693)	Minorities (*n* = 686)	Han Chinese (*n* = 378)
Jamar dynamometer				
dominant hand, mean	31.8 ± 7.4	20.8 ± 5.5	26.0 ± 8.0	22.1 ± 7.7
mean value for six times	32.2 ± 7.0	20.1 ± 5.3	26.3 ± 7.8	22.7 ± 7.7
dominant hand, max	33.6 ± 7.5	22.2 ± 5.7	27.6 ± 8.3	23.5 ± 7.9
maximal value for six times	36.0 ± 7.2	23.7 ± 5.7	29.2 ± 8.4	25.7 ± 8.4
Camry EH101 dynamometer				
dominant hand, mean	32.3 ± 7.1	21.1 ± 4.8	25.5 ± 8.0	24.2 ± 7.3
mean value for six times	31.7 ± 6.8	20.8 ± 4.6	27.0 ± 8.3	23.7 ± 7.2
dominant hand, max	33.9 ± 7.4	22.5 ± 5.0	25.1 ± 7.7	25.5 ± 7.4
maximal value for six times	34.9 ± 7.1	23.1 ± 4.9	27.7 ± 8.2	26.2 ± 7.6

### Reliability and measurement bias

**Fig. 2:**
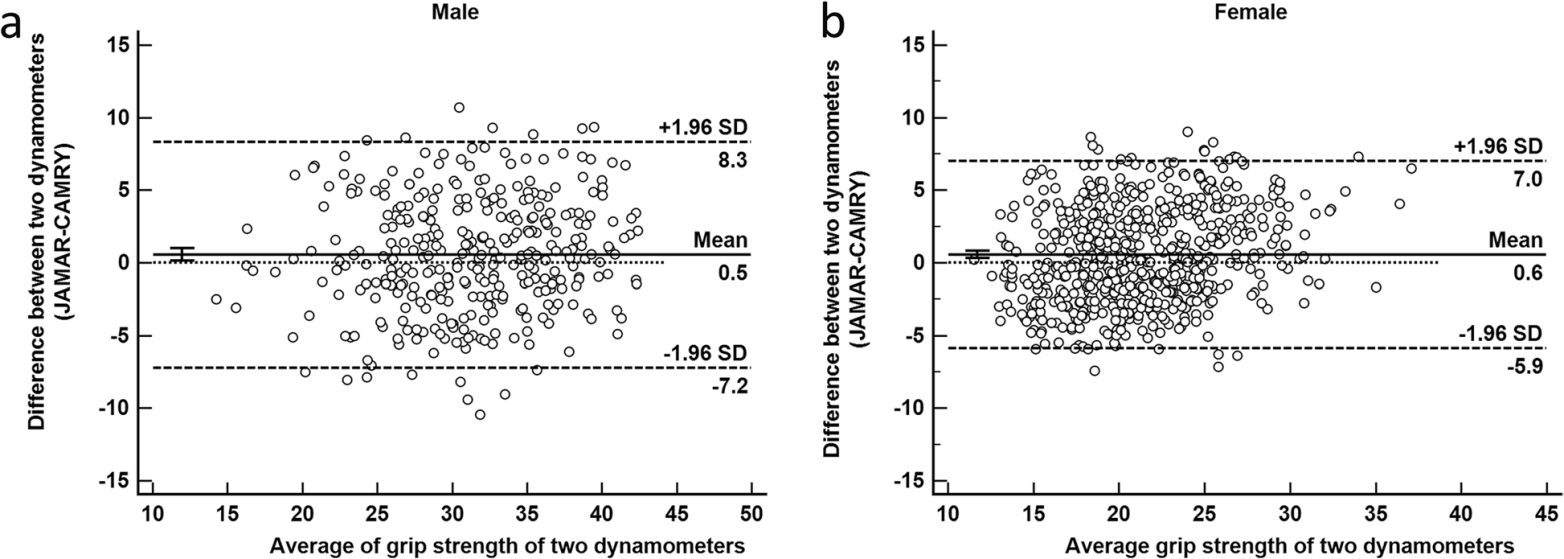
Bland–Altman plot comparing two dynamometers in men and women

**Fig. 3:**
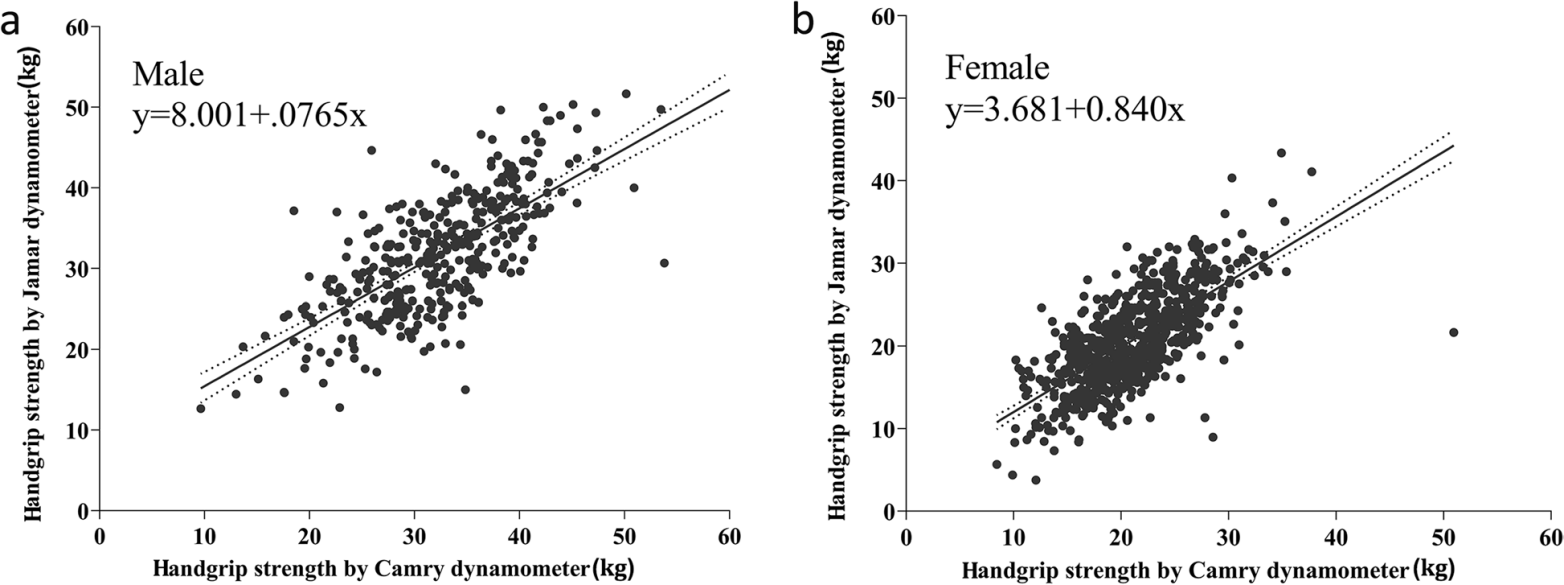
Linear regression comparing two dynamometers in men and women

To ensure generality and accuracy, we mainly analysed the average HGS six times. Outlier samples and and missing values were removed from the analysis. First, the ICC values between the two devices were 0.815–0.854, and the Spearman rank correlation of both dynamometers was 0.810–0.855 (Table 3), which indicated excellent reliability between the two dynamometers. Absolute reliability can be quantified using SEM and MDC. MDC can help clinicians evaluate whether the change between two measurements is a true change or just a measurement error. SEM was between 1.59–2.15 kg, and MDC ranged from 4.43 kg to 5.95 kg. The paired t test in this study showed a systematic bias between the two devices (p = 0.006). According to the Bland–Altman plot, systematic bias (Fig. 2a and b) underestimated by the CAMRY dynamometer was 0.5 kg in men and 0.6 kg in women compared with the Jamar dynamometer. To further explore the relationship between the two dynamometers and apply it in clinical practice, we carried out a linear regression equation by sex: male HGS (kg)_Jamar_ = 8.001 + 0.765 × HGS(kg) _CAMRY_; female HGS (kg) _Jamar_ = 3.681 + 0.840 × HGS (kg) _CAMRY_. (Fig. 3a and b).

**Table 3:** Reliability and validity of two handgrip dynamometers

	Jamar (kg) Mean ± SD	CAMRY(kg) Mean ± SD	ICC (95%CI)	r	SEM	SEM%	MDC	MDC%
dominant hand, mean	24.6 ± 8.1	25.0 ± 7.8	0.826 [0.806–0.844]	0.821	1.96	7.87%	5.44	21.86%
dominant hand, max	26.2 ± 8.4	26.4 ± 8.0	0.815 [0.794–0.835]	0.810	2.15	8.60%	5.95	23.79%
mean value for six times	25.0 ± 7.9	24.6 ± 7.5	0.854 [0.836–0.870]	0.855	1.59	6.40%	4.43	17.84%
maximal value for six times	28.0 ± 8.5	27.2 ± 8.0	0.844 [0.822–0.863]	0.852	1.81	6.54%	5.04	18.22%

r, speraman correation analysis; SEM, standard error of measurement; MDC, minimum detectable change

## Discussion

Asian expert consensus on sarcopenia recommends using a spring-type dynamometer to measure handgrip strength. CAMRY dynamometer, belongs to spring type dynamometer, is the most often used device to measure handgrip strength in Chinese studies. To the best of our knowledge, this is the first report on the relationship between the Jamar and CAMRY dynamometers. First, the relative reliability between the two dynamometers expressed by ICC varied from 0.815 to 0.854. Similarly, Spearman correlations between Jamar and the CAMRY dynamometer were 0.810–0.855. SEM% and MDC% were calculated to validate the precision and absolute reliability. Generally, SEM variability lower than 10% is considered appropriate for clinical purposes [14]. Other scholars insist that SEM% < 15% and MDC% < 30% demonstrate acceptable reliability [15]. Fortunately, the SEM% varied from 6.54% to 8.60%, and the MDC% varied from 17.84% to 23.79% in our research, which indicated high absolute reliability for the CAMRY dynamometer. The CAMRY dynamometer underestimated systematic bias in the Bland–Altman plots by 0.5 kg in men and 0.6 kg in women. As expected, the CAMRY dynamometer had excellent reliability and accuracy compared with the gold standard device. In summary, enough evidence has been shown from this review to recommend the use of a CAMRY dynamometer for Asian populations in clinical practice.

Evidence has confirmed that arm circumference, age, arm length, hand size, sex, height, and weight greatly influence HGS [16]. Generally, men had higher grip strength than women because of gender and physiological perspectives, and our findings are in accordance with this finding. Some scholars believe that HGS is significantly different between Caucasian and Asian populations. Marzetti et al. [17] investigated muscle strength and calf circumference among Italian and Taiwanese participants in 2018 and showed that Italian participants had significantly greater HGS and calf circumference than Taiwanese participants. Moreover, we comprehensively assessed HGS values for minorities living in highland areas and the Han Chinese population, and we observed that minorities had significantly higher HGS in both dynamometers. This phenomenon may be related to their distinct lifestyles, diet-related habits, living environment, culture, and genetic backgrounds [18].

The frequency of HGS testing and the interval between measurements remain controversial. Opinions about whether HGS tests should be measured twice or three times are always diverse and complicated; the same situation applies to average or maximal values. A systematic review in 2018 comprehensively analysed 34 epidemiologic studies and summarized that most studies carried out two or three trials; only one test was less reliable than three trials [19]. The ASHT recommends that the grip strength test be repeated at least three times each hand, and an average of three trials be selected for analysis [5, 20]. Fortunately, our study design fully complied with this principle. On the other hand, the HGS interval between measurements was inconsistent among current studies. Wu et al. [21] investigated older Taiwanese individuals in 2014 and reported that a 30-s interval was necessary; however, other literature recommended a 6-s break [22]. There was literature comparing constant measurement and 1-min rest and concluded that continuous measurement led to reduced power, but a 1-min interval counteracted fatigue effects [23]. In short, the ASHT believes that an interval of more than 15 s is reasonable for HGS testing procedures [20].

HGS values by the Jamar dynamometer were higher than those by the CAMRY dynamometer under the same circumstances in our research. Mathiowetz et al. [24] reported that HGS with the elbow in 90° flexion was higher than fully extended, but Lee et al. [25] investigated HGS in different positions of elbow and shoulder flexion at 90°, and 180° came to different conclusions. Kim et al. [8] found that the HGS of the Jamar hydraulic dynamometer was higher than that of the Smedley dynamometer. Expert consensus in AWGS 2019 also agreed on this viewpoint. In summary, hand size, measurement postures, joint position, and frequency of testing greatly influence the absolute values and precision of HGS, which makes comparisons between different devices difficult [5].

Most studies in this field have reported numerous types of hand dynamometers to measure grip strength in addition to Jamar, such as the DynEX, Grip-ball, Smedley, and other measurement devices. Kim et al. [8] reported evidence between the Jamar and Smedley dynamometers in 2017; 478 participants attended the study. There was systematic bias with underestimating HGS by the Smedley dynamometer compared with Jamar, bias 3.09 kg for men and bias 2.6 kg for women. Shechtman et al. [7] reported the reliability and validity of the digital DynEx Dynamometer in 2005. This study selected the Jamar criterion as the gold standard, and 100 young, healthy subjects aged 20–40 years were included; the data revealed high test–retest reliability for the DynEx Dynamometer (*r* = 0.9864) [7]. According to the present results, the sample size for the DynEx dynamometer is so small that more evidence is needed to support this conclusion. A grip-ball dynamometer can be used for home self-monitoring HGS because of a pressure sensor. Vermeulen et al. [26] reported that the Pearson correlations between grip ball and the Jamar dynamometer were 0.71 and 0.76 for the left and right hands, respectively. Indeed, the use of grip-ball dynamometers in our country is rare; on the other hand, the implementation of grip-ball as a screening and monitoring HGS device is uncertain.

This study had several limitations. First, the number of minorities living in the highland areas was significantly greater than that of Han Chinese. Second, 772 older adults had chronic disease among the 1064 community-dwelling adults; the relationship between chronic disease and grip strength changes is complicated. However, participants aged over 50 always have multiple comorbidities; therefore, a similar study must be performed once more in young, healthy volunteers.

## Conclusions

In the present study, HGS values by the Jamar dynamometer were higher than those by the CAMRY dynamometer. In summary, the CAMRY EH101 dynamometer provides excellent reliability and validity compared with the Jamar dynamometer. We concluded that the CAMRY dynamometer could serve as a reliable, inexpensive and practical device to assess grip strength in geriatric clinical practice.

## Data Availability

The datasets used for the current study are not publicly available due to confidentiality constraints but are available from the corresponding author upon reasonable request.

## Abbreviations

ICC: Intraclass correlation coefficients
HGS: Handgrip strength
AWGS: Asian Working Group for Sarcopenia
WCHAT: West China Health and Aging Trends Study
ASHT: American Society of Hand Therapists
SOP: Standard operation procedure
MUAC: Mid-upper arm circumference
CC: Calf circumference
SD: Standard deviation
SEM: Standard error of measurement
MDC: Minimal detectable change
LOA: Limits of agreement
